# Isolation, characterization and application of a cellulose-degrading strain *Neurospora crassa* S1 from oil palm empty fruit bunch

**DOI:** 10.1186/s12934-014-0157-5

**Published:** 2014-11-11

**Authors:** Qingxin Li, Wei Ting Ng, Jin Chuan Wu

**Affiliations:** Institute of Chemical & Engineering Sciences, Agency for Science, Technology and Research, 1 Pesek Road, Jurong Island, Singapore, 627833 Singapore

**Keywords:** Oil palm empty fruit bunch, Cellulose degradation, Biofuel, Cellulose

## Abstract

**Background:**

Oil palm empty fruit bunch (EFB) is a lignocellulosic waste produced in palm oil industry. EFB mainly consists of cellulose, hemicellulose (mainly xylan) and lignin and has a great potential to be reused. Converting EFB to fermentable sugars and value-added chemicals is a much better choice than treating EFB as waste.

**Results:**

A cellulase-producing strain growing on oil palm empty fruit bunch (EFB) was isolated and identified as *Neurospora crassa* S1, which is able to produce cellulases using EFB as the sole carbon source. The strain started to secret cellulases into the medium after 24 h of cultivation at 30°C and reached its maximal cellulase activity at 240 h. Mass spectroscopy (MS) analysis showed that more than 50 proteins were secreted into the medium when EFB was used as the sole carbon source. Among them, 7 proteins were identified as putative enzymes associated with cellulose degradation. The whole cell culture of *Neurospora crassa* S1 was used to hydrolyze acid-treated EFB, giving a total sugar yield of 83.2%, which is comparable with that (82.0%) using a well-known cellulase producer *Trichoderma reesei* RUT-C30 (ATCC56765).

**Conclusion:**

*Neurospora crassa* S1 is a commercially promising native cellulase producer for EFB hydrolysis especially when the sugars obtained are to be fermented to products that require use of non-genetically engineered strains.

## Background

Lignocellulose is one of the most abundant renewable resources produced in agriculture and forest industries [1] and is of great importance for human being in terms of its potential to be converted to fuels and chemicals [2]. Cellulose, the major component of lignocellulose, is a polymer consisting of glucose units that are connected to each other by β-1-4 glycosidic bonds [3]. In lignocellulosic wastes, the cellulose fibers are embedded in a matrix of hemicellulose and lignin, which protects cellulose from being degraded by enzymes under certain conditions [2]. Conversion of lignocellulosic wastes to value-added chemicals is attracting more and more attention because of the rapid depletion of fossil fuels and increasing demand for energy, food, chemicals and sustainable development. Extensive studies have been conducted to convert lignocellulosic wastes to biofuels and chemicals [4,5]. Two steps are needed to convert lignocellulosic wastes to sugars. The first step is the pretreatment of lignocellulosic material to release cellulose for easier attachment by enzymes. The second step is cellulose degradation by cellulases to release sugars [4,5], which can be further converted to value-added chemicals by microbial fermentation or chemical conversions.

Enzymatic degradation of cellulose has been studied for decades. There are mainly three types of cellulases, endoglucanase (1, 4-D-glucan-4-glucanohydrolase), exocellobiohydrolase (1, 4-D-glucanglucohydrolase or cellobiohydrolase) and β-glucosidase (β-D-gluoside glucohydrolase) [4,6,7]. The complete hydrolysis of cellulose needs the synergistic action of these three types of enzymes [4,7]. Many microorganisms including bacteria and fungi have been demonstrated to produce cellulases. The production of cellulases is an inducible and finely controlled process [8]. Enzyme production is usually initiated when cellulosic substrates are present. This is the reason why most cellulase-producing strains were isolated from environments rich in lignocellulosic wastes. The production of cellulases by microorganisms is a complicated process; strains induced by different substrates might produce different cellulases which are more suitable for acting on the specific substrates [7]. Therefore, although there are many cellulase-producing strains that are commercially available, it is still necessary to isolate new strains for more efficient degradation of specific lignocellulosic materials.

Oil palm empty fruit bunch (EFB) is a lignocellulosic waste produced in palm oil industry especially in Malaysia and Indonesia [9]. The annual EFB production in these two countries is estimated to be 12 million tons. EFB mainly consists of cellulose, hemicellulose (mainly xylan) and lignin. Converting EFB to fermentable sugars and value-added chemicals is a much better choice than simply treating EFB as a waste [10,11]. It has been shown that EFB can be converted to fermentable sugars by combined use of dilute acids and cellulases [11,12]. The combined use of sulfuric acid and phosphoric acid has been shown to have a synergistic effect for hydrolysis of EFB than using either of them alone [9,11]. We have succeeded in obtaining a total sugar yield of 82.0% from EFB by the combined use of dilute acids and whole cell culture of *Trichoderma reesei* RUT-C30, which is a well-known cellulase-producing strain but not specific to EFB [12]. Many commercially available strains have been modified through mutagenesis to achieve high cellulase activity [13]. It will be useful to screen a native strain from EFB to produce fermentable sugars; this would make it attractive to some industries which strictly prohibit the use of genetically modified organisms (GMOs), such as food industry. Therefore, we tried to isolate suitable fungal strains directly from those growing on EFB and obtained one strain which is able to grow fast using EFB as the sole carbon source. Here we report the isolation, characterization and application of this new isolate for EFB hydrolysis.

## Results

### Strain isolation

**Figure 1:**
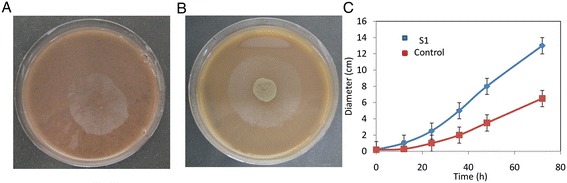
**Pictures of** ***Neurospora crassa*** **S1 (A) and** ***Trichoderma reesei*** **RUT-C30 (B) on EFB agar plates and the time courses of the fungal diameter (C).**

Many cellulase-producing strains have been isolated from the places rich in cellulose, which may be due to the fact that cellulase production is an inducible process. Fungal growth on EFB was observed when EFB was kept in humid environment, indicating that the fungi may use EFB as the sole carbon source for their growth. The fungi were isolated and tested for their ability to degrade EFB. It was found that the fungi isolated from EFB can grow in the agar plates containing EFB as the sole carbon source, suggesting their ability to degrade EFB (Figure 1). Strain S1 was shown to grow fastest. We analyzed the 18S rRNA of S1 and the result showed that its sequence is identical to *Neurospora sp* (Table 1). *Neurospora sp*. S1 (Figure 1C) showed a much higher growth rate on EFB agar plates compared to *Trichoderma reesei* RUT-C30 (ATCC 56765) which was used as control, suggesting that *Neurospora sp*. S1 might be a more suitable strain for converting EFB to sugars.

**Table 1:** **18S rRNA analysis of the screened strain**

**Description**	**Max score**	**Max identity**	**Accession**
Sordariomycetes sp. 5.8S rRNA	863	100%	JX298884.1
Neurospora intermedia 5.8S rRNA	863	100%	JX045846.1
Neurospora crassa 18S rRNA	863	100%	JX198494.1
Neurospora sp. 18S rRNA	863	100%	GU183173.1
Neurospora intermedia 18S rRNA	863	100%	EF197071.1
Neurospora intermedia 18S rRNA	863	100%	AY681192.1
Neurospora intermedia 18S rRNA	863	100%	AF388923.1

### Cellulase production

**Figure 2:**
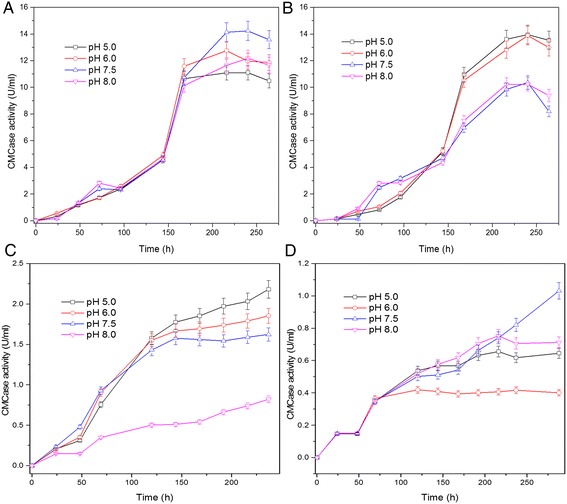
**Time course of cellulase production.** Cellulase production of *Neurospora crassa* S1 **(A)** and *Trichoderma reesei* RUT-C30 **(B)** using Avicel as carbon source is shown. Cellulase production of *Neurospora crassa* S1 **(C)** and *Trichoderma reesei* RUT-C30 **(D)** using EFB as carbon source is shown.

The cellulase production by *Neurospora sp*. S1 was tested using both EFB and Avicel as the carbon sources. Although it is well known that the components of the cultural medium affect the enzyme production, extensive medium optimization needs to be conducted to obtain the best medium for enzyme production. We used EFB and Avicel as the carbon sources for cellulase production of several strains during EFB treatment. We then compared the cellulase activity of stain S1 at different pHs because pH is one of the most important parameters that affect cellulase production. S1 cellulase activity (CMCase) reached maximum (14 U) at 213 h (Figure 2A) when using Avicel as the carbon source, which is similar to the reference stain (Figure 2B). When EFB was used as carbon source, S1 showed double the CMCase activity and grew faster compared to the reference strain (Figure 2C, D). Although these two strains exhibited different CMCase activities in different media (Figure 2), our result indicated the strong ability of *Neurospora sp*. S1 in degrading EFB, which contained lignin and hemicellulose in addition to cellulose. To produce cellulase using Avicel as sole carbon source for subsequent enzymatic hydrolysis of EFB using whole cell cultures, we selected the optimal pH for each strain that is pH 7.5 and 5, for S1 and ATCC 56765 respectively (Figure 2A, B).

### Identification of enzymes produced by *Neurospora sp*. S1

**Figure 3:**
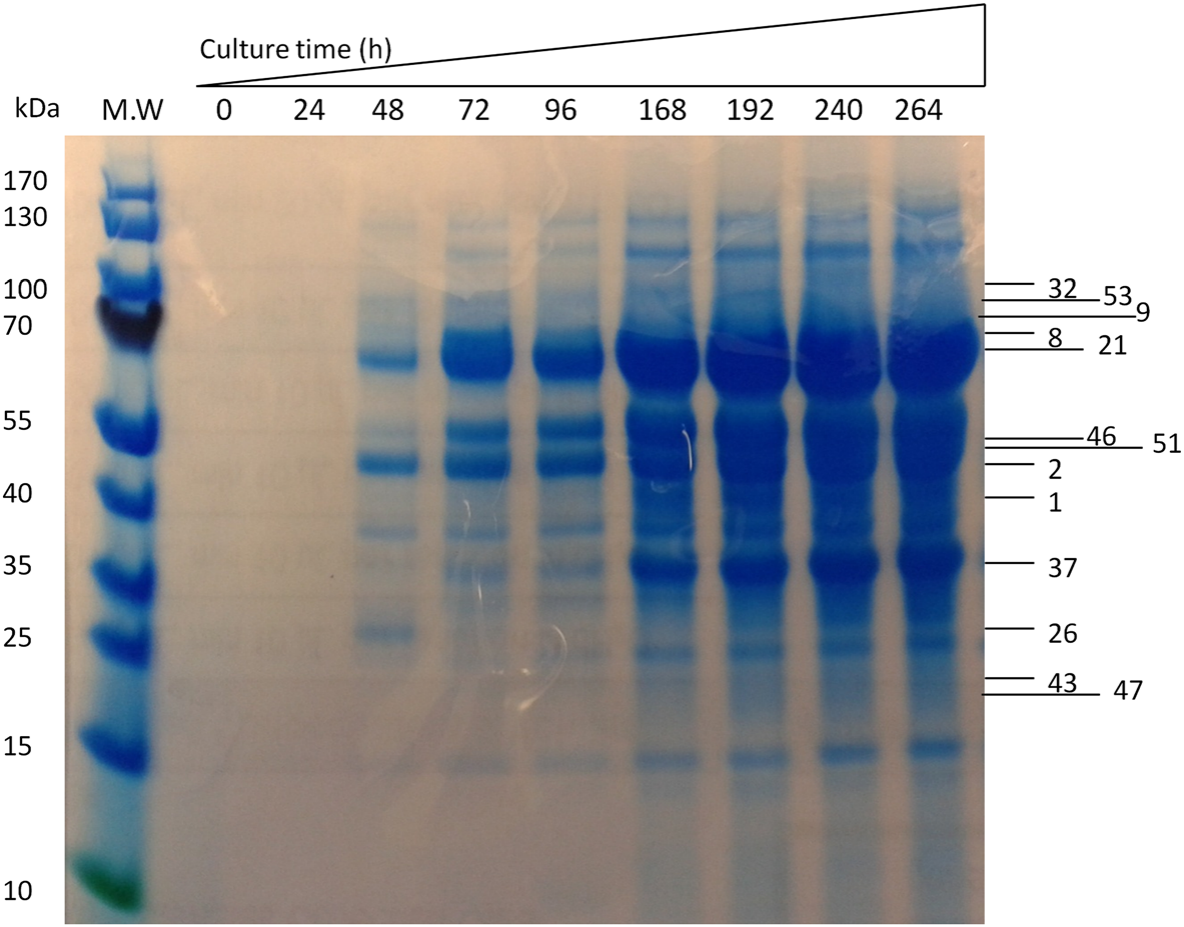
**SDS-PAGE analysis of the proteins secreted into the liquid medium with EFB as the sole carbon source.** The medium was taken out and concentrated before mixing with SDS-PAGE loading dye. The samples were heated at 100°C for 3 min then loaded onto SDS-PAGE gel. The protein bands were visualized by Coomassie Blue staining. The numbers listed on the left side of the gel are the numbers of proteins listed in Table 1. The upper panel of the gel shows the cultivation time of *Neurospora crassa* S1.

*Neurospora sp*. S1 culture was subjected to SDS-PAGE analysis to identify the proteins produced when EFB was used as the sole carbon source. S1 started to secrete proteins into the medium after 24 h (Figure 3), which is in good agreement with the occurrence of cellulase activity (Figure 2A, C). There are numbers of proteins secreted into the medium during strain growth as seen from the SDS-PAGE (Figure 3). To identify these proteins, we cut the whole lane of gel and extracted the proteins from the gel. The secreted proteins were identified by MS analysis. Over 50 proteins were identified from the protein mixture (Table 2). All these protein sequences matched with those of *Neurospora crassa,* strongly suggesting that the isolated strain S1 is *Neurospora crassa*. To understand the functions of the secreted proteins, the identified proteins were further compared with the genome of *Neurospora crassa* (http://www.broadinstitute.org/annotation/genome/neurospora/MultiHome.html). Among them, 7 proteins might be important for cellulase degradation (Table 2). Based on 18S rRNA sequence, the S1 can only be identified as *Neurospora sp*. From the MS results, S1 can be further identified to species because all the secreted proteins were identified as the proteins from *Neurospora crassa*. Although most of the secreted proteins have their predicted functions based on the information from the *Neurospora* genome, among them, 7 proteins have enzymatic activities that might be important for cellulose degradation including glycosylhydrolase (GH) 5–1, GH7-1, GH3-4, GH61-1, GH61-5, GH61-7 and endoglucanase IV (Table 2) which explained the CMCase activity measured in this study. Other enzyme such as β-glucosidase was also found to be secreted by S1. Our enzymatic activity assay also showed that the S1 product contained approximately 0.2 U/ml of β-glucosidase activity when EFB was used as a carbon source, further supporting the MS result.

**Table 2:** **Proteins secreted into the medium are identified by MS**

**No**	**Protein name**	**Predicted function**	**M.W**	**No**	**Protein name**	**Predicted function**	**M.W**
***1***	GH5-1	Endoglucanase	41.9	29	HP	NA	26.5
***2***	GH7-1	Endoglucanase	47	30	HP	NA	84.7
3	CHD-1	Cellobiose dehydrogenase	88.4	31	Dipeptidyl peptidase	Glutamyl cycle	75
***4***	GH61-5	Cellulose degradation	34.2	32	GLA-2	Glycogen degradation	115.3
5	GPI	Polysaccharide metabolism	43.1	33	Acw-12	NA	42.4
6	Cel74A	Polysaccharide metabolish	89.1	34	Α-Larabinofuranosidase	NA	72.6
7	Lectin 2B	Cell wall	16.3	35	Acw-7	NA	25
8	GH47-5	Carbohydrate metabolism	77.9	36	ebG	NA	31.8
9	Aminopeptidease 2	Peptide metabolic process	101.4	37	Tca-15	Malate metabolic process	35.5
***10***	GH3-4	Cellulose degradation	77.7	***38***	Endoglucanase IV	Cellulose degradation	25.8
11	TRE-1	Carbohydrate metabolism	77.5	39	Secreted protein	NA	22.2
12	eBG	NA	78.7	40	GPI-anchored eBG	Cell wall	41.9
13	Gllutaminase A	Glutamine degradation	73.2	41	3-phytase A	NA	66.5
14	CRO	NA	110.7	42	HP	NA	118
15	CWG	Cell wall	49.2	43	Lysome	Defense related protein	24.2
16	HP	NA	30.1	44	Glucuronan lyase A	NA	28.4
17	App	Cell-cell adhesion	23.8	45	HP	NA	34.4
18	6-phosphogluconolactonase	Pentose phosphate pathway	41.2	46	Alpha-N-arabinofuranosidase	Carboxylate catabolism	53.2
19	Tripeptidyl-peptidase	Peptide degradation	63.3	***47***	GH11-1	Xylan degradation	23.8
***20***	GH61-1	Cellulose degradation	32.8	48	Chitin deacetylase	NA	35
21	Beta-glucosidase	Beta-glucosidase	96.6	49	HP	NA	51.4
22	eBM	Carboxylate catabolism	44.9	50	Gh28-1	Polygalacturonase	38
23	HP	NA	36.1	51	oxdC	Oxalate decarboxylase	51.1
24	HP	NA	42.2	52	γ-glutamyltranspeptidase	Glutamyl cycle	64
***25***	GH61-7	Cellulose degradation	33.3	53	Gh35-1	Lactose degradation	108.6
26	RA	Polysaccharide degradation	28.1	54	HP	NA	20.6
27	HP	NA	99.5	55	HP	NA	18.1
28	Serine peptidase	NA	61.5	55	Beta-1,3-xoglucanase	Polysaccharide degradation	96.2

GH, glycosylhydrolase. eBG, exo-beta-1,3-glucanase. CRO, copper radical oxidase. CWG, Cell wall glucanase. APP, abundant perithecial protein. eBM, endo-beta-1,4-mannanase. RA, Rhamnogalacturonan acetylesterase. Gla-2, glucoamylase-2. Acw-12, anchored cell wall protein-12. Tca, tricarboxylic acid-15. HP, Hypothetical protein. NA, function not known. Cellulose degrading enzymes are highlighted in bold.

### Conversion of pre-treated EFB to fermentable sugars by *Neurospora crassa* S1

The combination of acid-catalyzed treatment of EFB and enzymatic hydrolysis of treated EFB using whole cell cultures has been shown to be an efficient way for converting EFB to fermentable sugars [12]. In our previous study, the whole cell culture of the reference strain, *Trichoderma reesei* RUT-C30 (ATCC 56765), was used for the cellulose hydrolysis. Here we used *Neurospora crassa* S1 to replace this commercial strain for the EFB hydrolysis under the same conditions as used before. When the S1 product containing same CMCase activity as the reference stain was used to treat EFB, the total sugar yield reached 83.2%, which is almost the same with that (82.0%) obtained using the reference strain [12]. The hydrolysate of EFB catalyzed by the whole-cell culture of *Neurospora crassa* S1 contained mainly glucose (59%) and xylose (4.3%). Although there were still approximately 20% of EFB sugars that could not be obtained by the treatment, *Neurospora crassa* S1 is as good as the well-known commercial cellulase-producer *Trichoderma reesei* RUT-C30 for hydrolyzing EFB. Further optimization of the whole-cell cultural conditions might improve the sugar yield. It is worth mentioning that *Trichoderma reesei* RUT-C30 is a hyper-secreting mutant of the wild type *Trichoderma reesei* QM6a for production of cellulolytic enzymes and recombinant proteins [13], which might be not suitable for some applications where the use of native strains are needed.

## Discussion

Extensive studies for screening and characterizing cellulose-degrading strains including bacteria and fungi have been conducted. Most of the strains were isolated from places rich in cellulose or from soil samples [14,15]. Studies have also been conducted to obtain recombinant cellulose-degrading enzymes [16]. Here we isolated a native *Neurospora crassa* S1 from EFB waste rich in cellulose. Our study showed that the isolated stain could produce enzymes with a CMCase activity of 14 U in liquid culture, similar to that of *Trichoderma reesei* RUT-C30 (ATCC 56765) used as a control when Avicel was used as carbon source. When EFB was used as a carbon source, the CMCase activity of S1 was higher (Figure 2). Many strains including the reference strain have been shown to have high ability to degrade cellulose, but most commercially available strains have been modified through mutagenesis [13]. EFB is a lignocellulose waste rich in cellulose and can be hydrolyzed to fermentable sugars which can be further converted to value-added chemicals for use in food or cosmetic industries [11]. It is thus necessary to screen a native strain from EFB to get fermentable sugars for use in food and cosmetic industries where the use of genetically modified strains is strictly prohibited. The isolated *Neurospora crassa* S1 can grow and produce cellulases when EFB was used as the sole carbon source. The growth of *Neurospora crassa* S1 is faster than that of *Trichoderma reesei* RUT-C30 in both liquid and solid cultures (Figures 1 and 2). It is worth mentioning that although higher cellulase activity was observed for *Neurospora crassa* S1 than the reference strain when EFB was used as carbon source, the experimental conditions used here may not be optimal for production of cellulases [12]. Although the CMCase activity of *Neurospora crassa* S1 was not very high compared to that of other cellulase-producing strains [17], the cellulase produced in the whole cell culture of *Neurospora crassa* S1 was still able to efficiently convert pretreated EFB to fermentable sugars. This further supports the hypothesis that cellulose degradation is a complicated process with many enzymes involved synergistically. We tried to use purified cellulases but failed to get higher EFB conversion than using the whole-cell cultures (data not shown). When hydrolyzing acid-treated EFB using the whole cell cultures, *Neurospora crassa* S1 and the reference strain gave almost the same total sugar yields [12], indicating the promising commercial applications of *Neurospora crassa* S1.

*Neurospora crassa* is a model cellulolytic fungus with its regulation of cellulose-degrading enzymes being tightly controlled [18-20]. Over 10 cellulose-degrading enzymes have been shown to be secreted into the medium induced by cellulose substrates [21]. Among the proteins secreted into the medium by *Neurospora crassa* S1 when EFB was used as the sole carbon source, 7 of them are proteins that are related to cellulose degradation (Table 2). Interestingly, the enzymes are different from those produced when pure cellulose was used as the carbon source [18]. In a previous study, glycoside hydrolases (GH) such as GH6-2, GH5-1, GH3-4, GH6-3, GH7-1, GH61-1, GH61-2 and GH61-5 have been shown to be secreted into the medium when *Neurospora crassa* was grown in a different medium. For *Neurospora crassa* S1, only GH5-1, GH7-1, GH61-1, GH61-5 and GH3-4 were found to be screened into the medium. Two cellulose-degrading related enzymes including endoglucanase IV and GH11-1 were found to be secreted by *Neurospora crassa* S1. These differences might be caused by *Neurospora crassa* S1 itself or by different inducers, which need further investigation. A recent study showed that some of the cellulose-degrading enzymes may not be dependent on inducers [22]. Our results indicate that different inducers during enzyme production might affect the gene expression of cellulose-degrading enzymes. Therefore, when preparing the whole cell cultures, using EFB as the inducer is more suitable for getting tailor-made cellulases for more efficient hydrolysis of EFB [22].

## Conclusions

*Neurospora crassa* S1 is able to produce cellulases using EFB as the sole carbon source. There were several cellulose-degrading enzymes secreted into the medium. The whole cell cultures of *Neurospora crassa* S1 could efficiently convert EFB to fermentable sugars. Therefore, *Neurospora crassa* S1 is a commercially promising strain for EFB hydrolysis especially when the sugars thus obtained are to be used for applications where the use of native strains is a must.

## Materials and methods

### Materials

EFB was obtained from Wilmar International Limited Corporation and sun-dried before grinding to 1 mm. The EFB powder was then dried at 80°C overnight before use. All chemicals used in this study were purchased from Sigma.

### Strain isolation

Fungi natively growing on EFB was placed in 100 ml medium containing 2 g/L (NH_4_)_2_SO_4_, 0.2 g/L MgSO_4_, 2 g/L KH_2_PO_4_, 1 g/L K_2_HPO_4_, 0.5 g/L NaCl, 1 (v/v)% corn steep liquor and 0.1% (v/v) trace element solution at pH 6.5. The trace element solution contained: 0.1 g/L ZnSO_4_.7H_2_O, 0.01 g/L H_3_BO_3_, 0.01 g/L Na_2_MoO_4_.2H_2_O, 0.1 g/L CoCl_2_.6H_2_O, 0. 1 g/L CuSO_4_.5H_2_O, 0.1 g/L FeSO_4_.7H_2_O, 0.5 g/L MnSO_4_.4H_2_O and 0.1 g/L CaCl_2_.2H_2_O. The strain was cultured for 3 d at 30°C and 150 rpm before being streaked on EFB agar plates containing 20 g/L EFB, 20 g/L agar, 100 mg/L ampicillin, 10 mg/L erythromycin, 50 mg/L kanamycin sulfate, 30 mg/L chloramphenicol and 10 mg/L tetracycline hydrochloride at pH of 6.0. The fungal colonies obtained on agar plates were streaked repeatedly to obtain pure cultures. Finally the fungal strains were obtained and maintained on potato dextrose agar (PDA) plates (4 g/L potato extract, 20 g/L dextrose and 20 g/L agar) for further studies.

### Strain identification

The genomic DNAs of the isolates were isolated and amplified using internal transcribed spacer 1 (ITS 1, 5′ -TCC GTA GGT GAA CCT TGC GG - 3′) and internal transcribed spacer 4 (ITS 4, 5′ -TCC TCC GCT TAT TGA TAT GC - 3′) primers. The amplified gene products were separated by agarose gel electrophoresis and purified using PCR purification and gel extraction kits. The amplified products were sequenced and the sequences were analyzed using BLASTN program (http://www.ncbi.nlm.nih.gov/).

### Cellulase production by isolated fungi

The cellulase production of the isolates and the reference strain ATCC56765 were investigated using Avicel and EFB as substrates at various pHs (5.0, 6.0, 7.5 and 8.0). The medium contained 2 g/L (NH_4_)_2_SO_4_, 0.2 g/L MgSO_4_, 2 g/L KH_2_PO_4_, 1 g/L K_2_HPO_4_, 0.5 g/L NaCl, 1 (v/v)% corn steep liquor and 20 g/L Avicel or EFB (1 mm). The pH was adjusted to the desired values prior to autoclaving. The strains were inoculated into 100 ml Avicel or EFB medium from PDA plates and incubated at 30°C, 200 rpm for several days. The cellulase activity was regularly measured during fungal growth.

### Enzymatic activity assay

Caboxylmethylcellulase activity was measured at 50°C and the released sugars were analyzed using a dinitrosalicylic acid method (DNS assay). The DNS reagent was prepared by dissolving 21 g of NaOH in 300 ml water followed by addition of 6.3 g of 3,5-dinitrosalicyclic acid. Then 237.2 g of sodium potassium tartrate tetrahydrate, 5 g of phenol and 5 g of Na_2_SO_3_ were dissolved and the solution was topped up to 1 L. Sodium caboxylmethylcellulose (CMC-Na) was used as a substrate [23] and was prepared by dissolving 1% (w/v) of carboxylmethylcellulose Na in 100 mM acetate buffer at pH4.8. The DNS assay was carried out by adding 0.1 ml of diluted fungal culture supernatant to 1 ml CMC-Na reagent followed by vortexing and incubation at 50°C for 30 min. The reaction was stopped by adding 3 ml of DNS reagent and incubation at 100°C for 5 min. The UV absorbance at 540 nm was detected. One unit of cellulase activity (U) is defined as the amount of enzyme that liberates 1 μmol of glucose per minute under the assay conditions [17]. The β-Glucosidase was assayed using p-nitrophenyl-β-D-glucopyranoside (pNPG) as substrate. One unit of β-glucosidase activity is defined as the amount of enzyme that is required to release 1 μmol of p-nitrophenol per minute under the assay conditions [24].

### MS analysis of proteins produced by *Neurospora sp.* S1

*Neurospora sp*. S1 was grown in aforementioned medium until the cellulase activity reached maximum. Then the culture was collected and centrifuged at 13,000 g for 10 min. The supernatant was concentrated using a concentrator with a molecular weight cut-off of 3.0 kDa. The concentrated solution was mixed with SDS-PAGE sample loading dye and separated by SDS-PAGE. To identify proteins that were secreted by the strain, the whole lane containing protein bands in the SDS-PAGE was collected. The gel containing the protein mixtures was dried, the proteins were extracted and digested with trypsin and identified by Tandem mass spectrometry. The identified proteins were compared using Blast analysis (http://www.ncbi.nlm.nih.gov/). The obtained sequence was further compared with the *Neurospora* genome using the blast tool provided by the website. (http://www.broadinstitute.org/annotation/genome/neurospora/MultiHome.html).

### Conversion of EFB to fermentable sugars by combination of acid-catalyzed treatment and enzymatic hydrolysis using whole cell culture of *Neurospora sp*. S1

Acid-catalyzed treatment of EFB was carried out as described previously [6] using the acid solution containing 0.5% (w/v) H_2_SO_4_ and 0.2% (w/v) H_3_PO_4_. The mixture (300 ml, 5% (w/v) EFB dry mass) was kept in a 1 L Parr reactor at 160°C for 10 min followed by rapid cooling down to room temperature. The solid fraction (cellulose-lignin complex) was collected by filtration for subsequent enzymatic hydrolysis using whole cell cultures of *Neurospora sp*. S1. *Trichoderma reesei* RUT-C30 (ATCC 56765) as a control strain and cultivated under the same conditions. The total sugar yield was calculated as a percentage of the total sugars obtained from both acid-catalyzed and enzymatic hydrolysis steps against the total sugars contained in EFB which could be released if hydrolysis was 100% efficient.

## Abbreviations

SDS-PAGE: Sodium dodecyl sulfate polyacrylamide gel electrophoresis
EFB: Oil palm empty fruit bunch
